# Clinimetric properties of hip abduction strength measurements obtained using a handheld dynamometer in individuals with a lower extremity amputation

**DOI:** 10.1371/journal.pone.0179887

**Published:** 2017-06-22

**Authors:** Ruud A. Leijendekkers, Gerben van Hinte, Amy D. Sman, J. Bart Staal, Maria W. G. Nijhuis-van der Sanden, Thomas J. Hoogeboom

**Affiliations:** 1 Department of Orthopaedics, Physical Therapy, Radboud University Medical Centre, Nijmegen, the Netherlands; 2 Radboud Institute for Health Sciences, IQ Healthcare, Radboud University Medical Centre, Nijmegen, the Netherlands; 3 Research group Musculoskeletal Rehabilitation, HAN University of Applied Sciences, Nijmegen, the Netherlands; 4 Department of Rehabilitation, Radboud University Medical Centre, Nijmegen, the Netherlands; Universitatsklinikum Aachen, GERMANY

## Abstract

**Introduction:**

Suitable handheld dynamometer (HHD)-techniques to test hip abduction strength in individuals with a lower extremity amputation, irrespective of their amputation level are absent. The aim of this study was to optimise a HHD-technique and to test its reproducibility and validity.

**Methods:**

This study involved three phases, in which two techniques were evaluated. Both HHD-techniques used a lever-arm of 22 centimetre. HHD-technique 1 used a break-technique. After obtaining within-session test-retest reproducibility (phase 1) we optimised the HHD-technique by adding a fixation-belt and using a make-technique (HHD-technique 2). We tested the within-session test-retest and inter-rater reproducibility (phase 2) and the validity (phase 3) of HHD-technique 2 using an isokinetic dynamometer. New cohorts of participants were recruited for each phase.

**Results:**

Phase 1: we tested HHD-technique 1 in 26 participants with a lower extremity amputation. It was test-retest reproducible (ICC3.1_agreement_: 0.80–0.92, standard error of measurement (SEM): 3.1–4.4 Nm and smallest detectable change (SDC): 8.6–12.3 Nm). There were questions regarding the validity of the measurement, because the mean muscle torque of the residual limb and sound limb were similar, which is uncommon. Phase 2: reproducibility of HHD-technique 2 was tested in 44 participants with a lower extremity amputation. It was test-retest reproducible (ICC3.1_agreement_: 0.96–0.97, SEM: 3.9–4.7 Nm and SDC: 10.9–12.9 Nm) but not inter-rater reproducible despite having good reliability (ICC3.1_agreement_: 0.92, SEM: 6.9–7.6 Nm and SDC: 19.2–21.2 Nm). Systematic bias and bias related to the magnitude of the muscle torque was suspected. Phase 3: the concurrent validity was established in 30 healthy participants (r = 0.84). Systematic bias in measurement error was present, including a consistent overestimation of the muscle torque of 28% using the HHD.

**Conclusion:**

HHD-technique 2 is a test-retest reproducible and valid measuring technique The technique may be further optimised by the use of an external device to stabilise the HHD.

## Introduction

Lower extremity muscle strength training is an important element of rehabilitation programmes for individuals with a lower extremity amputation[1–6]. The importance is supported by the following findings: 1) strength of the muscles of the hip joint in the residual limb is decreased up to 35% relative to healthy subjects and up to 28% compared to the sound limb [7,8]; 2) muscle atrophy of the residual limb is present up to 73% compared to the sound limb [9]; 3) decreased and asymmetric muscle strength is associated with lower gait speed and an asymmetric gait pattern [10–13] 4) decreased strength of the muscles of the hip joint is associated with lower activity levels [7]. Reliable, valid and responsive measurement instruments are needed to measure muscle strength. This is important to be able to determine the intensity of strength training and to evaluate the effectiveness of a rehabilitation programme.

In scientific research various instruments are used to evaluate lower extremity muscle strength in individuals with a lower extremity amputation, such as isokinetic dynamometers [4,7,8,14], an Optical Testing of Isometric Moments (OpTIMo) device [13], 10-repetition maximum tests on resistance machines [1] and handheld dynamometers (HHD) [3,15]. For daily clinical practice a measurement has to be low in cost, non-time-consuming, portable and easy to use, which is only the case for HHD measurements [16]. Clinimetric properties for muscle strength measurement obtained with a HHD are mainly established in able-bodied persons, but are lacking for individuals with a lower extremity amputation [16].

Various measurement techniques to evaluate hip abduction strength using a HHD are described in the current literature [17–27]. None of these measurement techniques is suitable for individuals with a transfemoral amputation because of the positioning of the HHD in relation to the absence of a knee or ankle joint. The main variations in execution are characterised by: 1) the participants’ position (side-lying or supine position), 2) the position of the HHD (slightly proximal to the edge of the lateral femoral condyle or the lateral malleolus, respectively), 3) the use of additional fixation-belts, 4) the type of resistance technique used (‘break-technique’ or ‘make-technique’), 5) the use of additional portable devices to stabilise the HHD [17–27].

**Fig 1:**
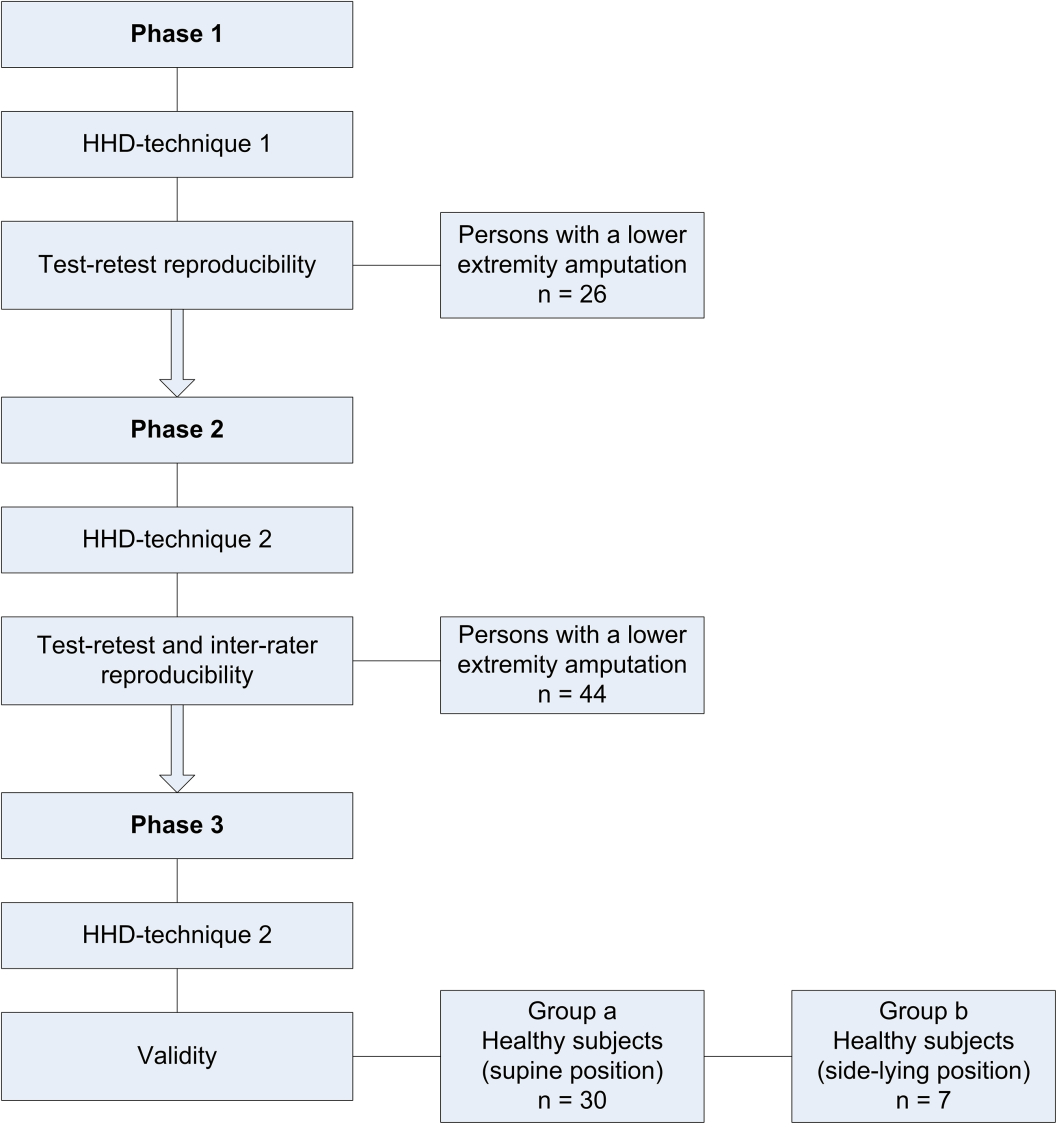
Flowchart study.

The aim of this cross-sectional study was to optimise a hip abduction strength measurement technique for individuals with a lower extremity amputation, irrespective of their level of amputation, and to test its reproducibility and validity. The optimisation of the HHD measurement technique in this study involved three phases (Fig 1), in which two techniques were evaluated. In both HHD-techniques the muscle strength of the participant was assessed in supine position, a gravity neutralised position [22], to prevent measurement bias due to weight differences between the residual limb and sound limb. Additionally, measurement variation is reduced by testing in a supine position compared to testing in a side-lying position [24]. A new cohort of participants was recruited for each phase of the study (Fig 1). The COSMIN Checklist was followed for the preparation of the manuscript [28].

## Phase 1: Test-retest reproducibility of HHD-technique 1

### Methods

The aim of this phase was to determine the within-session test-retest reproducibility of HHD-technique 1; a break-technique without the use of an additional fixation-belt or portable device to stabilise the HHD. We chose not to use any additional tools in order to make the test as practical as possible and to improve easy implementation in daily clinical practice.

#### Participants

**Fig 2:**
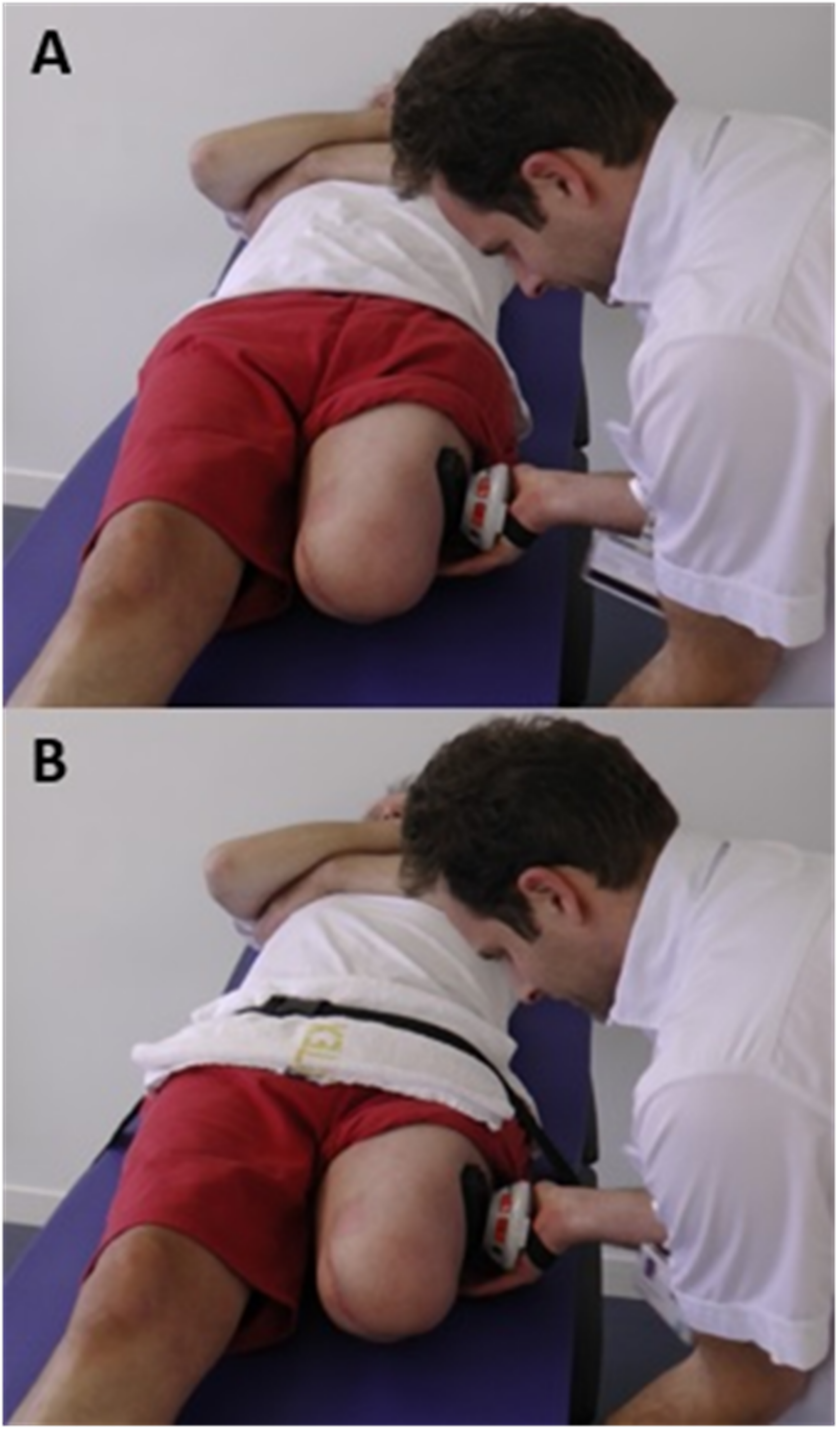
Assessment set-up. A: Handheld dynamometer-technique 1; B: Handheld dynamometer-technique 2, in default supine position.

All consecutive individuals within 3 months, with a lower extremity amputation who followed a rehabilitation program in our centre or had a regular follow-up were eligible for the study (Fig 1). A written informed consent was obtained from all participants prior to the assessment. The study was conducted according to the principles of the Declaration of Helsinki (64th version, 19-10-2013). The protocol of this phase of this study (registration number 2012/547) was approved by the Ethics Committees of the Radboud university medical centre. The individual displayed in Fig 2 has given written informed consent (as outlined in PLOS consent form) to publish this image.

#### Study procedure

The test-retest muscle strength assessment was performed by a physiotherapy student (RvE) following training from an experienced physiotherapist (RL). A pilot was performed where three individuals were tested using the measurement technique to ensure standardisation of the procedure. Both, the test and retest assessment were performed consecutively in one session on the same day. Both assessments started with muscle strength testing of the left limb followed by the right limb. The participants were offered sufficient time (at least 1 minute) to rest before the muscle strength test of each limb and between the assessments. The muscle strength value was not visible for the rater during the test as the screen of the HHD was positioned downwards (Fig 2A), to decrease the chance of measurement bias.

#### Testing procedure

Participants were positioned in supine position on a treatment table, which was covered with an additional anti-slip mat to prevent sliding (Fig 2A). The participants held their arms by their chest and the lower limbs were in neutral position (limbs shoulder-width apart). The rater placed a marking on the skin to indicate the point where the force would be applied. This mark was used for both the test and the re-test. The point was marked 20 centimetre (cm) distal of the most prominent aspect of the greater trochanter. When participants had a shorter residual limb, the lever-arm was adjusted and noted. Muscle strength was obtained in Newton (N) using the break-technique with a portable HHD (MicroFET2^TM^, Hoggan Scientific LLC., Salt Lake City, Utah, United States) including a 4 cm wide transducer pad. In this procedure, the rater applied a resistance that was sufficient to counteract the force generated by the participant, after which the rater gradually overcame the participants’ force and stopped the moment the limb gave way. The lever-arm to the centre of the pad (22 cm) was used to calculate the hip abduction torque value in Newtonmetre (Nm).

Following a warming-up of one submaximal contraction, all participants performed three maximal contractions for 3 to 5 seconds with a 1-minute rest interval for each limb [22,24]. The highest score of the three maximal contractions was used for analysis [24]. During all strength measurements, verbal encouragement was given [22].

#### Statistical analysis

Participant characteristics including sex, age, level of amputation and the length of the residual limb were described [29]. The residual limb length (cm) was measured from crotch to the most distal end of the residual limb. The torque values (Nm) were calculated for both the residual limb and the sound limb. The difference in muscle torque (Nm) between the test and the retest was calculated. Categorical data were presented as exact numbers and percentages were calculated for the various levels. For the continuous data, means and standard deviations were calculated.

Reproducibility (test-retest) was divided in reliability and agreement parameters [30]. Reliability was tested using the intraclass correlation coefficient (ICC). ICC’s were calculated using a two-way mixed effect model (ICC3.1_agreement_). with 95% confidence intervals (CI). The Interpretation of ICC values was based on guidelines offered by Byrt [31]: 0.01–0.20 poor reliability, 0.21–0.51 slight reliability, 0.41–0.60 fair reliability, 0.61–0.80 good reliability, 0.81–0.92 very good reliability, and 0.93–1.00 excellent reliability. Standard error of measurement (SEM_agreement_) and the smallest detectable change (SDC_agreement_) were calculated to assess agreement. Both are expressed in the unit of the measurement (Nm). The SEM was calculated as SEM_agreement_ = √σ^2^_error_ = √(σ^2^_o_+ σ^2^_residual_) [32]. The variance due to systematic differences between the observers (σ^2^_o_) and the residual variance (σ^2^_residual_) were obtained from the varcomp analysis [32]. The SEM_agreement_ was used to calculate the SDC_agreement_ = 1.96 * √n * SEM [30]. In this formula ‘n’ refers to the number of measurements, which is two in our study [30]. Additionally, the SEM % and SDC % were calculated as outcomes independent of the unit of measurement. The SEM % and SDC % were calculated by dividing the SEM and the SDC, respectively, by the average torque value of the test and the retest and then multiplying by 100 [21,23]. A Bland-Altman plot was constructed to determine if there was bias in measurement error [33,34]. This plot shows the rater difference against the mean muscle torque. The plot visualises the relationship between the measurement error and the observed value including the presence of systematic bias and bias related to the magnitude of hip abduction strength [34]. The 95% limits of agreement (95% LoA) were shown in the plot (mean difference ± 1.96 SD of the difference). All analyses were performed using IBM SPSS Statistics v22 (SPSS Inc., Chicago, Illinois, United States). In all cases, two sided p-values <0.05 were considered to be statistically significant.

### Results

We included 26 participants (20 men) with a lower extremity amputation (Table 1). The mean age of this group was 52 years (range: 24–80 years). We did not have to adjust the default lever-arm of 22cm in any of the included participants (n = 18) with a transfemoral amputation.

**Table 1:** Participant characteristics.

Participant characteristics	Phase 1		Phase 2		Phase 3			
	HHD-technique 1		HHD-technique 2					
					Group a		Group b	
	n = 26		n = 44		n = 30		n = 7	
Sex (male), n (%)	20	(77)	28	(64)	18	(60)	4	(57)
Age (yrs), mean (SD)	51.7	(15.0)	53.9	(12.7)	33.1	(15.6)	22.0	(1.9)
Amputation level								
- Transfemoral amputation, n (%)	18	(69)	35	(80)	NA		NA	
Length residual limb (cm), mean (SD)	21.4	(3.7)	21.1	(4.4)	NA		NA	
- Through knee amputation, n (%)	1	(4)	1	(2)	NA		NA	
- Transtibial amputation, n (%)	7	(27)	7	(16)	NA		NA	
- Foot amputation, n (%)	NA		1	(2)	NA		NA	

HHD: handheld dynamometer; Yrs: Years; cm: Centimetre; SD: Standard deviation

**Fig 3:**
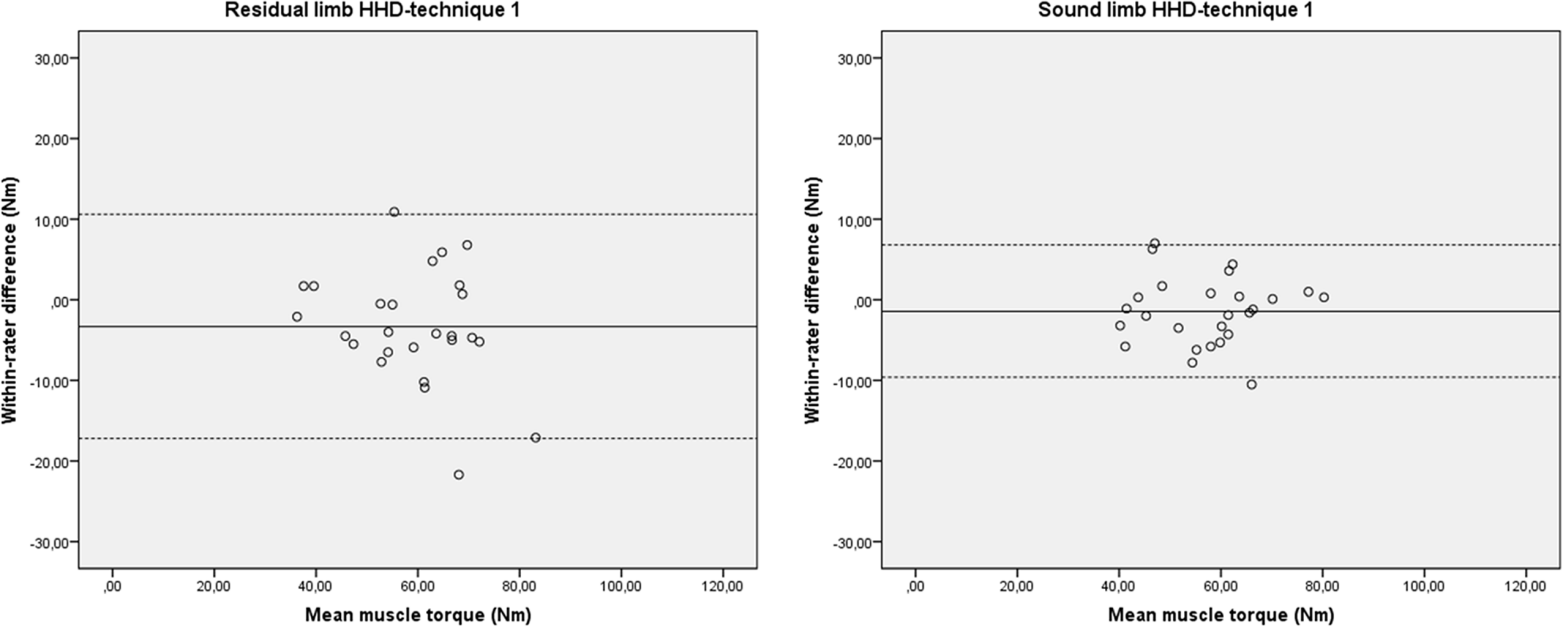
Bland–Altman plots for within-rater differences and their relation to the magnitude of hip abduction strength measured with HHD-technique 1. Nm: Newtonmetre; The solid line represents the mean difference (systematic bias) and the dashed lines illustrate the 95% limits of agreement (mean difference ± 1.96 SD of the difference).

The test-retest reproducibility of HHD-technique 1 is summarised in Table 2. We found fair to very good reliability (ICC3.1_agreement_: 0.80, 95% CI: 0.58–0.91) for the residual limb and very good to excellent reliability (ICC3.1_agreement_: 0.92, 95% CI: 0.83–0.97) for the sound limb. The SEM was 5.4 Nm and 3.1 Nm and the SDC was 15.1 Nm and 8.6 Nm in the residual limb and the sound limb, respectively. The SEM % was 9.1% and 5.4% and the SDC % was 25.5% and 15.0% in the residual limb and the sound limb, respectively. The 95% LoA was -17.2 to 10.6 Nm and -9.6 to 6.8 Nm for the residual limb and the sound limb, respectively (Table 2 and Fig 3).

**Table 2:** Phase 1: Test-retest reproducibility HHD-technique 1.

Tested limb	Test (Nm)	Retest (Nm)	Diff test-retest (Nm)	95% LoA (Nm)	ICC3.1_agreement_ (95% CI)	SEM_agreement_	SEM %	SDC _agreement_	SDC %
	mean (SD)	mean (SD)	mean (SD)			(Nm)		(Nm)	
Residual limb (n = 26)	57.5 (11.0)	60.8 (12.9)	-3.3 (7.1)	-17.2; 10.6	0.80 (0.58–0.91)*	5.4	7.4	15.1	25.5
Sound limb (n = 26)	56.5 (11.0)	58.0 (11.0)	-1.4 (4.2)	-9.6; 6.8	0.92 (0.83–0.97)*	3.1	5.4	8.6	15.0

HHD: handheld dynamometer; Nm: Newtonmetre; SD: Standard deviation; Diff: Difference; LoA: limits of agreement; ICC: Intraclass correlation coefficient; CI: Confidence interval; SEM: Standard error of measurement; SDC: Smallest detectable change

%: Percentage

*: p<0.001

#### Interpretation of the results

The reproducibility of HHD-technique 1 seemed good and no within-rater bias was present, but the results questioned the internal validity of the measurement. The mean hip abduction torque of the residual limb and sound limb were almost similar (Table 2), which did not correspondent with our observations during walking. Furthermore, these results were unexpected as no previous research reported on these [7,8]. In the residual limb, Ryser et al. [8] found a deficit of 28% in the hip abductor muscle torque and Kowal et al. [7] found a deficit of 15% in the hip extensor muscle torque compared to the sound limb. We identified two possible confounders which could have influenced the validity: 1) inconsistent participants’ fixation on the table because of differences between participants’ capacity to fixate themselves on the table with a residual limb or a sound limb, and 2) the relative high muscle strength values due to the use of the short-lever-arm (22 cm) and the break-technique [20]. High muscle strength values can also influence the participants’ fixation on the table and may have led to biased results because the strength of the rater will more likely influence the outcome [35]. Because of these findings and possible confounders we adjusted the HHD-technique for the next phase, resulting in HHD-technique 2.

## Phase 2: Test-retest and inter-rater reproducibility of HHD-technique 2

### Methods

The aim of this phase of the study was to determine the within-session test-retest and inter-rater reproducibility of HHD-technique 2. With this HHD-technique we strived to gather internally valid outcomes by decreasing the torque values and increasing the participants’ fixation on the table (Fig 2B). Therefore, we changed to the use of a make-technique and the use of additional fixation-belt. A potential advantage of using the make-technique is that it reduces the influence of the strength of the rater on the outcomes, whereas a break-technique produces higher torque values [36,37].

#### Participants

All consecutive individuals within 30 months, with a lower extremity amputation who were invited for a pre-operative assessment for a bone-anchored prosthesis between May 2014 and October 2016 were eligible for this part of the study (Fig 1) [38]. A written informed consent was obtained from all participants prior to the assessment. The study was conducted according to the principles of the Declaration of Helsinki (64th version, 19-10-2013). The protocol of this phase of this study (registration number 2014/196) was approved by the Ethics Committees of the Radboud university medical centre.

#### Study procedure

First, the test-retest assessments were performed by the first author (RL). Second, an experienced colleague (GvH) performed an additional assessment to test the inter-rater reproducibility. All assessments were performed consecutively in one session on the same day. All assessments started with muscle strength testing of the left limb followed by the right limb. The participants were offered sufficient time (at least 1 minute) to rest before the muscle strength test of each limb and between the assessments. The muscle strength value was not visible for the raters during the test as the screen of the HHD was positioned downwards (Fig 2B), to decrease the chance of measurement bias.

#### Testing procedure

The testing procedure of HHD-technique 2 was similar to HHD-technique 1, with the exception of the following: 1) a make-technique was used and 2) an additional fixation-belt at the level of the pelvis to fixate the participant on the table was used to prevent sliding (Fig 2B). This kind of fixation has previously been described by Pua et al. [22]. The make-technique involved a resistance, applied by the rater, that was sufficient to counteract the force generated by the participant. The participant was instructed to gradually increase the force aiming at a maximal contraction after 3 to 5 seconds.

#### Statistical analysis

Participant characteristics were calculated and presented in the same way as described in phase 1 of the study. The torque values (Nm) of both the residual limb and the sound limb were calculated. The difference in muscle torque (Nm) within the test-retest and within the inter-rater assessment was calculated. ICC’s were calculated using a two-way mixed effects model (ICC3.1_agreement_) with 95% CI for the test-retest reliability and using a two-way random effects model (ICC2.1_agreement_) for the inter-rater reliability [32,34]. The same parameters of agreement calculate in phase 1 (SEM_agreement_, SDC_agreement_, SEM % and SDC %) were calculated in this phase, for both the test-retest and the inter-rater reproducibility. The presence of bias in measurement error was assessed using the 95% LoA. All analyses were performed using IBM SPSS Statistics v22 (SPSS Inc., Chicago, Illinois, United States). In all cases, two sided p-values <0.05 were considered to be statistically significant.

### Results

We included 44 participants (28 men) with a lower extremity amputation (Table 1). The mean age of this group was 54 years (range: 27–78 years). In 3 out of 35 participants with a transfemoral amputation we had to adjust the default lever-arm from 22 cm to 18 cm.

**Fig 4:**
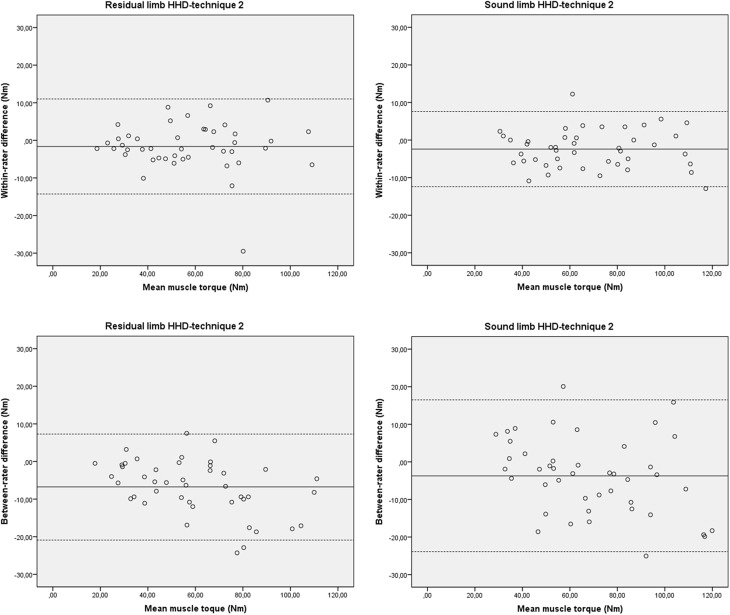
Bland–Altman plots for within-rater and between-rater differences and their relation to the magnitude of hip abduction strength measured with HHD-technique 2. Nm: Newtonmetre; The solid line represents the mean difference (systematic bias) and the dashed lines illustrate the 95% limits of agreement (mean difference ± 1.96 SD of the difference).

The test-retest reproducibility of HHD-technique 2 is summarised in Table 3. The test-retest reliability was excellent for both the residual limb (ICC3.1_agreement_: 0.96, 95% CI: 0.93–0.98) and the sound limb (ICC3.1_agreement_: 0.97, 95% CI: 0.94–0.99). The SEM was 4.7 Nm and 3.9 Nm and the SDC was 12.9 Nm and 10.9 Nm in the residual limb and the sound limb, respectively. The SEM % was 8.3% and 5.7% and the SDC % was 22.7% and 16.0% in the residual limb and the sound limb, respectively. The 95% LoA was -14.1 to 10.9 Nm and -12.4 to 7.6 Nm for the residual limb and the sound limb, respectively (Table 3 and Fig 4).

**Table 3:** Phase 2: Test-retest reproducibility HHD-technique 2.

Tested limb	Test (Nm)	Retest (Nm)	Diff test-retest (Nm)	95% LoA (Nm)	ICC3.1_agreement_ (95% CI)	SEM_agreement_	SEM %	SDC _agreement_	SDC %
	mean (SD)	mean (SD)	mean (SD)			(Nm)		(Nm)	
Residual limb (n = 44)	56.1 (22.9)	57.8 (23.3)	-1.6 (6.4)	-14.1; 10.9	0.96 (0.93–0.98)*	4.7	8.3	12.9	22.7
Sound limb (n = 44)	67.0 (24.5)	69.4 (24.6)	-2.4 (5.1)	-12.4; 7.6	0.97 (0.94–0.99)*	3.9	5.7	10.9	16.0

HHD: handheld dynamometer; Nm: Newtonmetre; SD: Standard deviation; Diff: Difference; LoA: limits of agreement; ICC: Intraclass correlation coefficient; CI: Confidence interval; SEM: Standard error of measurement; SDC: Smallest detectable change

%: Percentage

*: p<0.001

The inter-rater reproducibility of HHD-technique 2 is summarised in Table 4. The inter-rater reliability was fair to excellent for the residual limb (ICC2.1_agreement_: 0.92, 95% CI: 0.59–0.97) and very good to excellent for the sound limb (ICC2.1_agreement_: 0.92, 95% CI: 0.84–0.96). The SEM was 6.9 Nm and 7.6 Nm and the SDC was 19.2 Nm and 21.2 Nm in the residual limb and the sound limb, respectively. The SEM % was 11.6% and 11.0% and the SDC % was 32.8% and 30.8% in the residual limb and the sound limb, respectively. The 95% LoA was -20.9 to 7.3 Nm and -23.9 to 16.5 Nm for the residual limb and the sound limb, respectively (Table 4 and Fig 4).

**Table 4:** Phase 2: Inter-rater reproducibility HHD-technique 2.

Tested limb	Tester (Nm)	Tester 2 (Nm)	Diff test-retest (Nm)	95% LoA (Nm)	ICC2.1_agreement_ (95% CI)	SEM_agreement_	SEM %	SDC _agreement_	SDC %
	mean (SD)	mean (SD)	mean (SD)			(Nm)		(Nm)	
Residual limb (n = 44)	56.1 (22.9)	62.9 (26.1)	-6.8 (7.2)	-20.9; 7.3	0.92 (0.59–0.97)*	6.9	11.6	19.2	32.3
Sound limb (n = 44)	67.0 (24.5)	70.7 (27.9)	-3.7 (10.3)	-23.9; 16.5	0.92 (0.84–0.96)*	7.6	11.0	21.2	30.8

HHD: handheld dynamometer; Nm: Newtonmetre; SD: Standard deviation; Diff: Difference; LoA: limits of agreement; ICC: Intraclass correlation coefficient; CI: Confidence interval; SEM: Standard error of measurement; SDC: Smallest detectable change

%: Percentage

*: p<0.001

The measurements of both rater 1 as rater 2 identified an asymmetry in muscle torque between the two limbs. The muscle torque of the residual limb was 11 to 16% lower than the muscle torque of the sound limb.

#### Interpretation of the results

The test-retest reproducibility of HHD-technique 2 was good. The reliability had increased (ICC3.1_agreement_: 0.96–0.97 versus ICC3.1_agreement_: 0.80–0.92) and the SEM was similar (3.9–4.7 Nm versus 3.1–5.4 Nm), compared to HHD-technique 1 (Tables 2 and 3). The SDC of the residual limb was better (12.9 Nm versus 15.1 Nm) and the SDC of the sound limb was slightly worse (10.9 Nm versus 8.6 Nm), relative to HHD-technique 1. No within-rater bias was found, but there were suspicions for systematic bias and bias related to the magnitude of the muscle torque within the inter-rater test, in particular for the test of the residual limb. On average, the values of the second rater were higher than those from the first rater. The difference between raters increased when the subjects exhibited larger hip abduction strength (Fig 4).

We found a muscle torque deficit up to 16% in the residual limb compared to the sound limb. This is in line with previous research, where deficits of 15 to 28% are described [7,8]. Based on these results we were more confident that the internal validity of HHD-technique 2 was superior to HHD-technique 1. To test the internal validity of HHD-technique 2, phase 3 of this study was conducted.

## Phase 3: Concurrent validity of HHD-technique 2

### Methods

The aim of this phase was to determine the concurrent validity of HHD-technique 2 using an isokinetic dynamometer. In the HHD assessment the default participants’ position was a supine position, a gravity neutralised position [22], to prevent measurement bias due to different weight of the residual limb and sound limb. The default position for participants during the isokinetic dynamometer assessment was a side-lying position. This could not be changed, therefore this phase of the study involved two parts (Fig 1): 1) assessment of the concurrent validity of HHD-technique 2 in supine position and 2) assessment of the hip abduction strength using HHD-technique 2 in side-lying position. The aim of the second part was to rule out bias resulting from of the participants’ position on the table.

#### Participants

**Fig 5:**
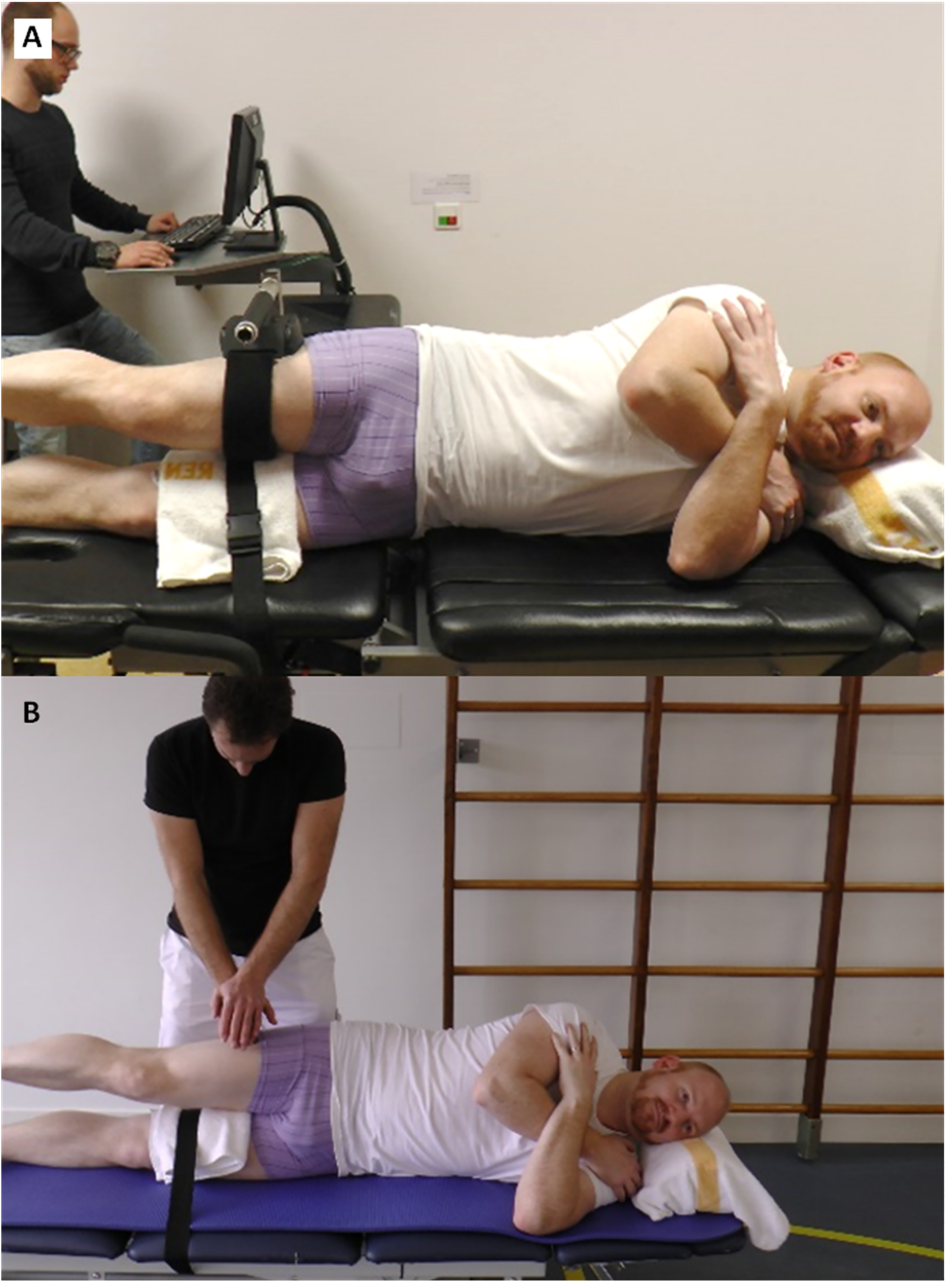
Assessment set-up. A: Humac Norm; B: Handheld dynamometer-technique 2, in side-lying position.

This phase of the study was conducted at the HAN University of Applied Sciences. All physiotherapy students of the HAN and their relatives were eligible for this part of the study. They were recruited within a time period of three months using posters, leaflets and social media. All included participants were assessed in supine position (group a). Twenty-five percent of these participants (group b) were randomly selected, based on their availability, to also perform the HHD assessment in side-lying position (Fig 1). A written informed consent was obtained from all participants prior to the assessment. The study was conducted according to the principles of the Declaration of Helsinki (64th version, 19-10-2013). The protocol of this phase of this study (registration number 2014/196) was approved by the Ethics Committees of the Radboud university medical centre. The individual displayed in Fig 5 has given written informed consent (as outlined in PLOS consent form) to publish this image.

#### Study procedure

The HHD assessment and the isokinetic dynamometer assessment were performed by trained physiotherapist students (respectively by YB and JD). A pilot, including three individuals, was performed to ensure standardised tested methods. In part 1, the participants (group a) were randomised into two groups using opaque sealed envelopes. One group started with the HHD assessment followed by the isokinetic dynamometer assessment. The other group had a counterbalanced programme in order to avoid bias as result of fatigue. In part 2, the participants (group b) performed an additional HHD assessment, in side-lying position, one week after the initial assessment.

Both assessments started with muscle strength testing of the left limb followed by the right limb. The participants were offered sufficient time (at least 1 minute) to rest before the muscle strength assessment of each limb and between the assessments.

#### Testing procedure

In part 1, HHD-technique 2 was used as previously described in phase 2. For comparison we obtain the isometric maximum voluntary contraction (Nm) of the hip abductor using an isokinetic dynamometer (Humac Norm, Computer Sports Medicine Inc., Stoughton, Massachusetts, United States). When using the Humac Norm, the participants were positioned in side-lying position with a fixation-belt on the thigh of the non-tested limb (Fig 5A). The stabiliser pad of the Humac Norm was positioned on the tested limb, 20 cm distal of the most prominent aspect of the greater trochanter. Following a warming-up of one submaximal contraction, all participants performed three maximal contractions of 3 to 5 seconds with a 1-minute rest interval for each limb [22,23]. The highest score was used for analysis [23]. During all strength measurements verbal encouragement was given [22].

In part 2, hip abduction strength using HHD-technique 2 in side-lying position was tested (Fig 5B). The position of the fixation-belt was adjusted so that it was similar to the fixation on the Humac Norm. The testing procedure was similar to the procedure described in part 1.

#### Statistical analysis

Participant characteristics including sex, age were calculated and presented in the same way as described in phase 1 of this study. The highest muscle torque values (Nm) of the left and the right limb were pooled. The difference in muscle torque values (Nm) obtained with HHD technique-2 and the Humac Norm were calculated. The concurrent validity between the muscle torque detected by HHD-technique 2 and the Humac Norm was determined by calculating the two-way mixed effects model (ICC3.1_consistency_) with 95% CI [39]. The presence of bias in measurement error was assessed using the 95% LoA. To determine bias resulting from the participants’ position on the table we analysed the muscle torque differences between the HHD-technique 2 in supine and side-lying position using the Wilcoxon signed-rank test. All analyses were performed using IBM SPSS Statistics v22 (SPSS Inc., Chicago, Illinois, United States). In all cases, two sided p-values <0.05 were considered to be statistically significant.

### Results

We included 30 healthy participants (group a), of which 18 men. The mean age of this group was 33 years (range: 20–64 years). The subgroup that was also assessed in side-lying position (group b) consisted of 7 participants (4 men), with a mean age of 22 years (range: 20–25 years) (Table 1).

**Fig 6:**
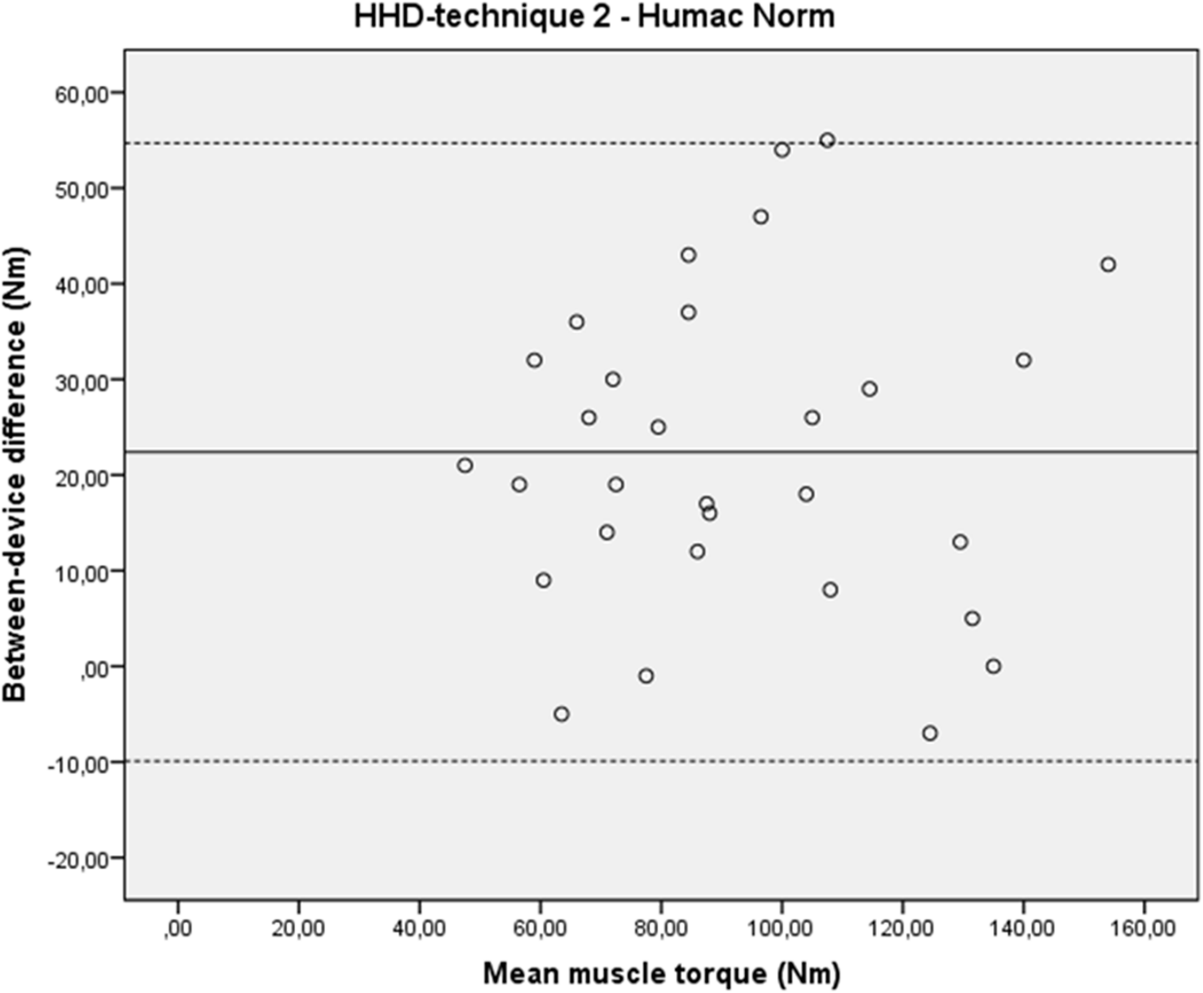
Bland–Altman plot for between-device differences and their relation to the magnitude of hip abduction strength measured with HHD-technique 2 and Humac norm. Nm: Newtonmetre; The solid line represents the mean difference (systematic bias) and the dashed lines illustrate the 95% limits of agreement (mean difference ± 1.96 SD of the difference).

Results of the comparison of HHD-technique 2 to the Humac Norm outcomes (Table 5) revealed that the concurrent validity was good to very good (ICC3.1_consistency_: 0.84, 95% CI: 0.69–0.92), but that there was a systematic bias in measurement error (Fig 6). Hip abduction torque measured with the HHD was 28% higher than when the muscle torque was measured with the isokinetic dynamometer. The 95% LoA was -9.9 to 54.7 Nm (Table 5 and Fig 6).

**Table 5:** Phase 3: Validity HHD-technique 2.

Type of instrument	Torque (Nm)	Difference HHD-Humac	95% LoA (Nm)	ICC3.1_consistency_
	mean (SD)	(Nm) mean (SD)		(95% CI)
HHD (n = 30)	103.7 (29.3)	22.4 (16.5)	-9.9; 54.7	0.84 (0.69–0.92)*
Humac Norm (n = 30)	81.3 (28.9)			

HHD: Handheld dynamometer; Nm: Newtonmetre; SD: Standard deviation; LoA: limits of agreement

*: p<0.001

The hip abduction torque of the sample (n = 7) that performed the HHD-technique 2 both in supine and in side-lying position was 107.1 (9.0) Nm and 104.1 (21.5) Nm, respectively. Comparison of these results showed that the participants’ position did not influence the outcome (p = 0.799). The hip abduction torque on the Humac Norm was 93.1 (31.9) Nm in this sample.

#### Interpretation of the results

The concurrent validity of HHD-technique 2 was good in healthy subjects and the participants’ position did not result in biased results. A systematic bias in measurement error was present. The muscle torque measurement with a HHD resulted in a consistent overestimation of the hip abduction torque compared to measurement with an isokinetic dynamometer. The mean overestimation was 28%.

## Discussion

The reproducibility of both presented HHD-techniques was good, however HHD-technique 1 may have resulted in less valid outcomes. This was illustrated by the absence of muscle torque asymmetry between the residual limb and sound limb. The second phase of this study revealed that an adjustment of HHD-technique 1, by adding a fixation-belt and changing the type of resistance (HHD-technique 2), may have led to internal valid outcomes. This was confirmed in phase 3 of this study. However, hip abduction torque measured by a HHD-technique 2 overestimated the muscle torque while the gold standard measurement revealed lower muscle torque values. Because the overestimation is consistent it is possible to convert values obtained with a HHD-technique 2 to isokinetic dynamometer values.

The test-retest reliability of HHD-technique 2 (ICC3.1_agreement_ 0.96–0.97) was similar as previously reported HHD test-retest reliability (ICC 0.74–0.98) [17,22,24,25] and intra-rater reliability (ICC 0.81–0.96) [18–21,26,27] of hip abduction strength measurements. The inter-rater reliability of HHD-technique 2 (ICC2.1_agreement_ 0.92) was better than outcomes reported in previous studies (ICC 0.58–0.88) [18,20,23,26,27]. These findings suggest that the test-retest and inter-rater reliability of HHD-technique 2 is sufficient. This means that, despite measurement errors, HHD-technique 2 is appropriate to distinguish individuals with a lower extremity amputation from each other [30]. Krause et al. [20] previously stated that a short lever-arm could negatively influence the level of reliability. This was not confirmed in our study as the results showed very good to excellent reliability for HHD-technique 2.

Agreement parameters for HHD measurements of hip abduction strength are scarcely described in the current literature [18,19,21–24]. This makes comparison difficult, partly because the SEM and SDC are reported in various units of the measurement. Newton [19,21,23,24], Newtonmetre [18,22,26] and kilogram [27] were used, but information concerning the exact lever-arm was missing hereby eliminating the possibility to recalculate the outcomes. In four studies [21,23,24,27] the SEM % and/or SDC % was described and, in three other studies [19,22,26] there was enough data available to calculate them. Because both the SEM % and SDC % are independent of the unit of measurement it was possible to compare our HHD-technique 2 results to these studies. The test-retest SEM % and SDC % of HHD-technique 2 ranged from 5.7 to 8.3% and 16.0 to 22.7%, respectively. These results are within the range found in the literature [19,22,24,26]; 2.9 to 13.7% for the SEM % and 8.1 to 31.7% for the SDC %. These findings combined with the absence of bias in measurement error, as illustrated in Bland and Altman plots (Fig 4), show that HHD-technique 2 is a valuable tool for test-retest repeated measurements to identify changes in hip abduction strength in patients with a lower extremity amputation. The inter-rater SEM % and SDC % of HHD-technique 2 ranged from 11.0 to 11.6% and 30.8 to 32.3%, respectively. The literature [21,23,26,27] shows similar values; 3.3 to 20.2% for the SEM % and 9.4 to 26.7% for the SDC %. The large inter-rater SDC % and the fact that the results raised suspicions for (systematic) bias in measurement error, also illustrated in Bland and Altman plots (Fig 4), limits applicability of HHD-technique 2 in an inter-rater clinical practice. A possible explanation for these results is the presence of variation in rater strength.

Between-rater bias as result of variation in rater strength is, particularly found in measurements of hip abduction strength with a short lever-arm [35]. The level of between-rater bias may be decreased with the use of an external device to fixate the HHD instead of stabilisation by a human rater [23]. Despite the use of an external device, less desirable results were found in short-lever set-ups than in long-lever set-ups.[23] The short-lever set-up used by Thorburg et al. [23], was a HHD placement just above the knee. This is much longer than the lever-arm we used (22 cm). Using external devices to stabilise the HHD could potentially increase both the test-retest as inter-rater agreement parameters of HHD-technique 2. Therefore, the use of external devices to stabilise the HHD within HHD-technique 2 is worthwhile to explore, but the use of a longer lever-arm is not an option for individuals with a transfemoral amputation.

The concurrent validity of HHD-technique 2 (ICC3.1_consistency_: 0.84, 95% CI: 0.69–0.92) is good to very good and is similar to the concurrent validity of other HHD-techniques used to obtain hip abduction strength [17,18]. Aramaki et al. [17] found a Pearson’s correlation coefficient of 0.82 in healthy young adults. Hebert et al. [18] found an ICC3.1 of 0.69 in healthy adolescents. On average, HHD-technique 2 produced a 28% higher hip abduction strength value than the isokinetic dynamometer. This illustrates that the instruments are not interchangeable. This is a problem when both instruments are used interchangeably within the evaluation of the muscle strength of one individual, but this is not likely in daily clinic. However, these findings must be taken into account when comparing different cohorts, as different research studies often use different testing methods. We did not use a gravity-correction within the analysis of the assessments in side-lying position. This may led to lower hip abduction torque values and is thus a possible confounding factor [40]. Previous research of the concurrent validity of manual muscle testing with a portable HHD revealed inconsistent results; higher [41], lower [42,43] but also similar magnitude [17,44–46] of the muscle strength have been described when compared to the assessment with an isokinetic dynamometer. Thus, the difference in muscle strength found in this study between the assessments with the HHD and the isokinetic dynamometer is not surprising. More important to note is that the difference was consistent in relation to the magnitude of the hip abduction strength (Fig 6). Therefore it is possible to convert values obtained with HHD-technique 2 to isokinetic dynamometer values.

### Strengths and limitations

An important strength of this study is that it resulted in a test-retest reproducible and valid measurement technique to evaluate hip abduction strength in patients with a lower extremity. A measurement technique like this was absent up to now. A second strength, is that we used a stepwise approach to optimise the HHD-technique where the findings of the first step were used to improve the HHD-technique. This approach resulted in the following insights: a fixation belt is of added value, an inter-rater setting is not desirable and the procedure can be standardised except for the lever-arm. Deviating from the default lever-arm of 22 cm is rarely needed, but when necessary it is possible to so because the unit of measurement is Nm. A third strength, is the sample size (26 to 44 participants) within the various phases of the study. The sample in this study is much larger than previous clinimetric studies regarding assessment of hip abduction strength [17–19,21–25,35], which ranged from 9 to 20 participants. Despite the lack of an a priori power calculation we are confident that our study was sufficient powered. Previous studies [22,23] that conducted an a priori power calculation determined that a sample size of 18 to 19 participants was necessary to achieve an acceptable ICC of at least 0.70.

A few limitations were identified. First, the point of force application was marked by the first rater on the skin one time and reused during subsequent assessments. A new point was marked only for the HHD test in side-lying position in phase 3, which was executed a week after the assessment in supine position. We chose to reuse the point marked to rule out differences in lever arm between the assessments. In a clinical practice with repeated measures, reusing the same marking point is not possible. This could lead to additional measurement variation of the within-rater and the between-rater results due to inconsistency in the placement of the force application. Second, the test-retest and inter-rater reproducibility was tested in one session on the same day which potentially could have resulted in recall bias of both the participant and the rater. Also the assessments to test the validity where in one session on the same day. The following reduced the chance on biased results: the muscle strength testing were alternated between the left limb followed by the right limb, sufficient time was taken between the assessments and the rater could not see the muscle strength value during the test. In clinical practice a retest of the muscle function is typically performed over a period of several weeks. However, in our study we assessed test-retest performance on the same day. We chose this procedure because the muscle strength of the included participants with a lower extremity amputation might have otherwise changed over time because the participants followed a rehabilitation programme (phase 1) or had surgery on a very short notice (phase 2). Whether test-retest performance is adequate over a longer period of time needs to be established in a stable population of patients. Third, changing HHD-technique 1 into HHD-technique 2 resulted in an asymmetry in muscle torque of the residual limb and sound limb which was expected [7,8], but the muscle torque did not decrease. We expected a reduction in muscle torque [36,37], because a make-technique instead of a break-technique was used in HHD-technique 2. We aimed to achieve lower muscle torque values because this may positively influence the participants’ fixation on the table and decrease the level of between-rater bias as result of variation in rater strength. A possible explanation for the absence of the muscle torque decrease may be that the true muscle strength of the participants in phase 2 was greater than that of the participants of phase 1 of this study. Another explanation may be the influence of the short-lever arm on the magnitude of the muscle torque. This may be so large that the effect of change in the type of resistance is irrelevant. Despite the high muscle torque values we did not find a ceiling effect in our study, illustrated by the Bland–Altman plots (Figs 3 and 4). To avoid a ceiling effect, which negatively influences the level of reproducibility, it is important that the strength of the rater is sufficient to overcome the strength of the participant. A possible advantage of the make-technique is that it is more comfortable and have shown a lower risk for injury than the break-technique [18]. Finally, HHD-technique 2 was validated within a healthy subject population while it was optimised for individuals with a lower extremity amputation. This could affect the generalisability of our results, because the magnitude of the muscle torque in healthy subjects is greater than that of individuals with a lower extremity amputation. However, the Bland and Altman plot (Fig 6) showed that the level of overestimation of the muscle torque by the HHD assessment was not related to the magnitude of the muscle torque. We do recommend that future studies include individuals with a lower extremity amputation to further validate HHD-technique 2.

## Conclusion

HHD-technique 2 is a valuable test-retest measurement technique to assess hip abduction torque in individuals with a lower extremity amputation. It is not recommended to use the test in a setting where the measurements are performed by various raters. The validity was established in healthy subjects. Future research could establish validity of the HHD-technique 2 in individuals with a lower extremity amputation and explore the potential advantage of incorporating an external stabilisation device to further optimise this technique.

## Supporting information

S1 File Data phase 1. (SAV)

S2 File Data phase 2. (SAV)

S3 File Data phase 3. (SAV)
